# Co‐Design of a Community of Practice for People With Personal and Professional Expertise or Interest in Dementia in Australia

**DOI:** 10.1111/hex.70503

**Published:** 2026-02-26

**Authors:** Eliza Watson, Sadia Afrin, Clarissa Giebel, Katrina M. Long, Jane Thompson, Matthew F., Chris Moran, Yen Ying Lim, Darshini Ayton

**Affiliations:** ^1^ School of Public Health and Preventive Medicine Monash University Melbourne Victoria Australia; ^2^ Department of Primary Care and Mental Health University of Liverpool Liverpool UK; ^3^ NIHR Applied Research Collaboration North West Coast Liverpool UK; ^4^ Department of Occupational Therapy School of Primary and Allied Health Care Monash University Frankston Victoria Australia; ^5^ National Centre for Healthy Ageing Frankston Victoria Australia; ^6^ Public Advisory Lead Australia; ^7^ Turner Institute for Brain and Mental Health, School of Psychological Sciences Monash University Clayton Victoria Australia

**Keywords:** caregivers, community of practice, dementia, health personnel, public and patient involvement and engagement

## Abstract

**Background:**

Communities of practice offer an opportunity to break down siloes for those with personal and professional experience of dementia. We aimed to design the Dementia Learning and Research Community in Australia, a virtual space to bring people with an interest or experience of dementia together to learn from each other, and provide opportunities for education and networking.

**Methods:**

A three‐phase co‐design process was conducted, consisting of six co‐design workshops (or two workshops per phase) with people with personal or professional experience of dementia. The workshops addressed the three elements of a community of practice—the community, domain and practice. Participants discussed their preferences for these elements through facilitated group discussions and were provided with summaries of key points after each workshop.

**Results:**

A total of 16 participants attended the workshops, including people living with dementia, carers, healthcare workers and researchers. They identified the need for a comfortable, respectful space that covers a range of research and care‐based topics. Participants suggested webinars, discussion forums, panel sessions and resource banks within a members' only online platform. They highlighted the need for platform accessibility for people living with dementia and those in rural areas. A public facing website and members' only platform have now been created.

**Conclusions:**

This co‐design process successfully brought together a range of participants with personal and professional experience of dementia to identify preferences for a community of practice on dementia across Australia. Future evaluations of the members' only platform will allow it to continue to evolve and identify areas for improvement.

**Patient and Public Contribution:**

A Public Advisory Committee (comprising one person living with dementia and three former carers of people living with dementia) were involved during the grant writing stage, reviewed research documents (including participant information sheets) and provided guidance on the co‐design workshop content. Two of the members of the committee who are former carers also reviewed this manuscript and are co‐authors.

## 1 Introduction

Learning in healthcare and research spaces is often siloed. Those from professional backgrounds, such as healthcare workers or researchers, are often unaware or don't recognise the importance of personal experiences of those living with a disease, their family members and current and former carers [1, 2]. Similarly, people with living experience of a disease and carers are often excluded from academic and healthcare spaces, leaving them unaware of developments around care and treatment or opportunities for involvement in research. This dynamic is further perpetuated by social norms that dictate roles and knowledge hierarchies between healthcare professionals, researchers and people directly impacted by a disease or health condition [1, 2].

People living with dementia and their carers face these issues during care and treatment, in addition to insufficient communication, lack of provision of post‐diagnostic information and perceptions of reduced capacity resulting in exclusion from healthcare conversations [3]. Progress has been made in improving involvement of people living with dementia and their carers in research, and the positive impact of this has been demonstrated [4, 5, 6]. However, more can be done to overcome perceived barriers to their involvement, and to assist researchers and others with professional expertise in dementia to learn about experiences of living with and caring for someone living with dementia [7].

Communities of practice (CoPs) offer an opportunity to break down siloes and bridge boundaries. They allow people with all types of expertise or interest—whether personal or professional—to engage with each other, increase collective knowledge, expand skills, network and improve their sense of belonging [8, 9, 10]. CoPs bring together groups of people with an interest in a particular topic to enable collective learning through shared activities and interactions over time [8, 11, 12]. They can focus on any number of topics, from professional development in business and healthcare sectors, to health condition support and education. CoPs were first defined in the 1990s as a learning theory that posits learning as a social process that takes place through participation in social practices outside of formal educational contexts [11, 12]. They can emerge spontaneously such as in professional contexts, or they can be developed and cultivated for example for support groups for those living with health conditions [8]. CoPs can have a variety of structures, and may involve online or face‐to‐face activities such as workshops, discussion sessions or journal clubs, depending on the intended purpose of the CoP. These activities are scheduled anywhere from weekly to quarterly, based on availability and preferences of the group members [13, 14, 15]. Effective design of a CoP requires working with potential community members, building on their in‐depth experience of the topic of interest to understand how learning should be approached in the community [8].

CoPs in the context of dementia have been developed for informal and formal caregivers, and people living with dementia. These CoPs have shown success in knowledge sharing and acquisition, creating trust and strengthening resilience [16, 17, 18]. Researchers in the United Kingdom have successfully used a CoP approach as a model for the Liverpool Dementia & Ageing Research Forum [15, 19, 20]. This forum is the first of its kind and brings together those with a personal *and* professional interest in dementia research and services, rather than focusing on those with personal experience alone. The Liverpool Dementia & Ageing Research Forum runs public seminars, webinars, journal clubs and annual conferences. It has resulted in hundreds of registrations for webinars, co‐produced research ideas and publications, opportunities for involvement in research, and has led to empowering those with personal experience in dementia in seeking out support and care that is available to them [15, 20]. We sought to develop a virtual CoP for dementia research and learning tailored for the Australian context called the Dementia Learning and Research Community (DLRC). The DLRC has been developed to provide education, information, networking and involvement opportunities for people with personal and professional experiences of dementia. We aimed to identify participants' preferences of elements for the Community using Wenger et al.'s CoP framework [8]. We employed Patient and Public Involvement (referred to as consumer and community involvement in Australia) principles and co‐design methodology to design a CoP that is suitable for a range of people with personal and professional interest and expertise in dementia in Australia.

## 2 Methods

### 2.1 Setting, Participants and Recruitment

This co‐design process was conducted online, based in Melbourne, the capital of Victoria, Australia. People with personal and professional expertise and interest in dementia Australia‐wide were invited to participate—including people living with dementia, current and former carers, healthcare and aged care workers, researchers, staff from not‐for‐profit advocacy and support organisations and advocates. Convenience sampling was used to invite participants who were in the investigator team's networks, with additional snowball sampling inviting those who were contacted to share the study with others who may be interested in participating. Participants were required to be 18 years or over and able to speak and read English due to the recruitment materials and workshops being in English, and to enable communication with other participants. We sought to recruit participants from a range of personal and professional experience groups to ensure a diversity of views. Participants were invited via email and completed an electronic consent form, were asked to describe their personal or professional experience or interest in dementia and nominated preferred times to attend the workshops.

Consideration was made regarding the inclusion of people living with dementia in the workshops. Participants were asked to self‐assess their capacity to participate, and were given the option to participate alongside a carer. It was anticipated that those with advanced dementia would opt‐out of participation. These decisions were based on previous experience working with people living with dementia and input from the Public Advisory Committee (outlined below) who also revised information, consent and recruitment documents.

### 2.2 Patient and Public Involvement

The project team was supported by a Public Advisory Committee consisting of four members: three former carers and one person living with dementia. Three of the members (including the person living with dementia) were involved during the grant writing stage, while the fourth member was recruited to the Committee after commencement of the project. All reviewed the concept of the community of practice, providing feedback and comments on the grant application, and one suggested the name ‘Dementia Learning and Research Community’. Following award of the grant, the Committee provided feedback and guidance on the protocol, recruitment materials including the participant information sheet and consent form and demographics data collection for participants, and the co‐design workshop content. Two carer members of the Committee have also reviewed this paper, providing comments and feedback on the content and wording, and ideas for inclusion in the discussion.

### 2.3 Community of Practice

CoPs share a unique structure of three fundamental elements: the community, domain and practice. These are defined below [8, 12, 21]:

- *Community (mutual engagement)*—The community is the group members and their relationship, their engagement with and support of one another. Building a strong sense of community fosters trust and mutual respect between members.
- *Domain (a joint enterprise)*—The domain is the shared interest that defines the community, brings participants together and spurs action. Defining the boundaries of the domain assists members in understanding what ideas and stories to share and affirms the purpose of the community.
- *Practice (a shared repertoire)*—The practice is the resources, frameworks, stories, information and tools that the community develops. Having a set of shared resources enables the community to function efficiently.

These elements can be used as a structural model for the development of a CoP, allowing for clearer definition of the purpose and collective action of the community [8]. We used this model to shape the development of the DLRC.

Wenger et al. [8] also describe seven principles for cultivating CoPs. These will be addressed in future publications during evaluation of the DLRC.

### 2.4 Study Design

This co‐design activity used Participatory Action Research, an approach which values experiential knowledge for collaboration and co‐creation [22]. We conducted a series of co‐design workshops to develop the community, domain and practice of the DLRC. Three virtual workshops were held via Zoom between September and October 2024. This format was chosen to allow for participants from across Australia to attend. The format was discussed with the Public Advisory Committee and it was felt this would provide the most accessible format, including for those living with dementia. The participant information sheet outlined the need for a device that could connect them to the internet and be used for video calls, and stipulated that a carer could be present to assist.

Each workshop was conducted twice (for a total of six workshops) to allow more participants to attend and to ensure the numbers of participants in each was not too large. Smaller group sizes were used to support participants living with dementia to be able to follow the content and discussion more easily. The content of the workshops is shown in Table 1, which has been mapped to the three elements of CoPs. One researcher (final author) led the workshops, another two researchers (first and second author) made notes and monitored questions. During the workshop introduction, participants were made aware of the different ways they could communicate with the group, for example by typing in the chat box, putting up their hand (literally or using the Zoom function), or by coming off mute. The researcher leading the workshops would prioritise those living with dementia in being able to respond to prompts first before other participants. The workshops were audio‐recorded and auto‐transcribed by Zoom. A summary of the discussions from each pair of workshops was sent to participants for their reference. This article provides a summary of the key points discussed in the workshops, including those which were raised or supported by more than one participant and those which generated discussion amongst the group.

**Table 1:** Workshop structure and content for the design of the Dementia Learning and Research Community and the corresponding community of practice elements.

Workshop	Item	Description	Community of practice element
1	Welcome and introduction	Acknowledgement of country and introduction of facilitator/s	
	Overview of communities of practice	Brief presentation to cover the principles of CoP and the overarching purpose of DLRC:	
		Consumer and community involvement in research and health and social care improvement. Knowledge exchange. A space for learning and asking questions of each other. Networking.	
	DLRC member needs	A facilitated discussion to understand:	Community
		What do community members need to engage and feel supported to engage in the community? What are their needs in the dementia research, care and support space? What would members like to achieve from being a part of the community?	
	Prompt for workshop 2	What kind of activities would members like as part of the DLRC?	
2	Welcome and introduction	As above.	
	Summary of workshop 1	What we heard and learnt about what people want and need.	
	DLRC proposed activities	A facilitated discussion to understand what activities members would like the DLRC to provide and the frequency of these.	Practice
	DLRC proposed topics	A facilitated discussion to understand what topics members would like to see being discussed and presented in the DLRC.	Domain
	Prompt for workshop 3	What resources and information would members require to engage with the DLRC?	
3	Welcome and introduction	As above	
	Summary of workshop 2	Summary of activity preferences.	
	DLRC proposed resources	A facilitated discussion to understand what resources and support members require to participate in the DLRC, including how to make the community accessible to members.	Practice and community
	Choosing a logo for the DLRC	Presenting participants with three logo choices for the DLRC.	Community

The DLRC website and member portal were developed based on the discussions in the workshops, incorporating elements that could be feasibly included at this time.

### 2.5 Ethics

This study was approved by the Monash University Human Research Ethics Committee (project ID 42316). All participants provided written informed consent to take part in the workshops.

## 3 Results

### 3.1 Participant Characteristics

A total of 16 participants consented to participate in the workshops. Thirteen attended workshop 1, 14 attended workshop 2 and 10 attended workshop 3 (combined totals of both iterations of each workshop). One participant withdrew after workshop two citing work commitments. Two participants (12.5%) attended one workshop, all others attended two (*n* = 7, 44%) or all three (*n* = 7, 44%) workshops. Participants were asked how they described their personal or professional experience or interest in dementia (Table 2). Other demographics were not collected. Key findings from the workshops are summarised below in the context of Wenger's CoP approach. In presentation of the results, participants refer to those who participated in this study; members refer to future members of the DLRC upon its inception.

**Table 2:** Participant self‐descriptions of their personal or professional experience of dementia.

How would you describe yourself?	Number^a^
A person living with dementia	2
Caring for or have cared for a person living with dementia	6
Advocate for or have an interest in dementia	5
Healthcare professional	6
Researcher or academic	5
Working in aged care	1
Working in another dementia related field	0
Other	1^b^

^a^ Participants were able to select more than one category to describe themselves.

^b^ Volunteer.

### 3.2 Community of Practice

#### 3.2.1 Community

The needs of CoP members are vital to its functioning and to ensure respectful and meaningful engagement. Workshop 1 focused on exploring these needs.

Participants wanted a space that was casual and non‐judgemental where they could share their stories and feel that they were being listened to, and where all members are treated equally regardless of title, profession, scope or status. They suggested introductions at the start of activities, or online written profiles to create a comfortable and familiar environment, and smaller groups of between 6 and 12 people for group discussions to assist with turn taking. Participants emphasised the need to be respectful of others' communication styles and to give space and time to members to express their thoughts, especially people living with dementia. The importance of effective facilitation was emphasised to ensure equal participation and encouragement to participate, for example through private direct messages.

Some participants felt that they would be hesitant to speak up in DLRC activities due to a fear of using incorrect terminology. It was highlighted that the purpose of the CoP is for learning and as such, participants should not be fearful of, or told off for, making mistakes. Discussing the group's preferred terminology, for example how group members would like to be referred to, may alleviate this fear.

Based on the discussion in workshop 1, the research team developed *Community Etiquette* guidelines. These were presented at the beginning of workshop 2, where the name was changed (originally titled *Rules of Engagement*) and wording and formatting was changed. The Public Advisory Committee and investigators also provided feedback. The finalised Community Etiquette is presented in Supporting Information 1.

Participants hoped to see tangible outcomes emerge from being members of the community and requested that results and outcomes be communicated with members. Current and former carers and family members of people living with dementia asked that their experiences and perspectives be heard and understood by those with professional experience of dementia. They described often feeling frustrated and dealing with a lack of support and asked for space and understanding from other members. Health and aged care workers and researchers agreed with this sentiment and expressed a willingness to learn more from those with personal experiences of dementia. Researchers also asked to receive resources to support effective involvement and engagement of people with personal experience of dementia in research.

Workshop 3 included a discussion of accessibility for community members. Participants supported use of an online platform, citing the difficulty in bringing people together in‐person from across Australia. Participants suggested a clean and simple online presence with minimal imagery and videos to assist both people living with dementia and those living rurally and regionally who may have limited internet connections. Participants suggested having options for those who would like to participate via different means, for example via phone and having hard copy documents available. Text size and spacing should be considered and phone‐ and tablet‐friendly versions of the community should be tested.

Participants were also presented with three options for the DLRC logo in workshop 3, designed by a graphic designer engaged by the research team. There was a robust discussion in both iterations of the workshop with most participants ultimately preferring one logo with some minor alterations due to its representation of intersecting groups and bringing people together. The final version of the logo was decided on with input from the Public Advisory Leads and the research team.

#### 3.2.2 Domain

The broad domain of the DLRC is dementia, however, determining the scope of this domain by understanding the special areas of interest for members will streamline the group's functioning. In workshop 2, participant preferences for topics were discussed, with additional discussion in workshop 3.

Participants discussed misconceptions of dementia in the media, by the general public and from some healthcare professionals. They felt it was important to bust these myths through scientific understanding of what dementia is and through personal stories and experiences of dementia. Participants from all personal and professional experience groups expressed interest in learning about all types of dementia related research, including basic science and discovery research, to clinical and therapeutic innovations and social and public health initiatives. Additionally, some wanted to improve their understanding of data and statistics surrounding dementia. Participants suggested having researchers and healthcare workers present their work in short, easy to understand presentations which use minimal jargon and are accessible to all participants. There was also discussion of promoting positive outcomes, with participants wanting to learn more about successful innovations in the broader community for improving the lives of people living with dementia, carers and families. Ethical issues in dementia research were also highlighted.

In terms of dementia care, participants (particularly those with personal experience of dementia) wanted to learn more about post‐diagnostic support, dementia care pathways and how to navigate them, nonpharmacological approaches to care and how to care for carers. Participants also discussed the importance of highlighting to members that the group's purpose is not to provide therapeutic support or individual advice to members.

#### 3.2.3 Practice

The practice represents the repertoire, tools and resources required to bring together the community and domain. Workshops 2 and 3 focused on understanding what the DLRC should look like, including activities and resources.

It was decided that a public facing website and members' only portal would be needed for the DLRC. The public facing website should provide information to potential members on what the community involves, why it was created, and a sign‐up page for the members' only platform, asking members for their name, email address and a screening question asking why they want to join the community. Collection of other personal details was discussed and it was decided that only these details should be collected, with members then able to share more within the members' only platform if they choose to do so. The members' only platform would then enable a private and safe space for members to share their thoughts, stories and opinions, and to share research and initiatives that may not be in the public domain. Within this platform, participants suggested having resource banks of important links for all participant groups, discussion forums, webinars, panel presentations and working groups. They highlighted the importance of having activities available in different formats (video and written) to suit different thinking styles.

Discussion forums would have prompts related to the topics outlined above and offer a space for participants to share their own experiences and reply to each other. Webinars may be offered on specific topics with recordings to be made available to members. Webinars may also provide opportunities for discussion between participants. In line with participants' preference for smaller groups, regular break out rooms were suggested to help develop relationships between members and to discuss topics more comfortably and confidently.

Participants supported the suggestion of panel presentations bringing together different perspectives, for example a researcher, a healthcare worker and a person living with dementia or a carer, to discuss their different experiences and views on a topic.

Dementia research was a key area of interest for all participants. Several ideas were suggested for bringing research ideas to members through presentations and discussions. These suggestions may be further developed as the DLRC evolves.

### 3.3 Development of the Dementia Learning and Research Community

Based on the workshop discussions and feedback from the Public Advisory Committee, we developed a public facing website and a members' only platform through which to access the DLRC. The website contains high level information about what the DLRC is, what members can expect to find in the DLRC and a sign‐up form [23]. Within the members' only DLRC platform, members of the community can access resource lists, discussion forums and links to and recordings of webinars and meetings that will take place via Zoom. An email address and phone number are available for direct access to the research team. Participants can request hardcopies of documents be sent to them. The chosen logo for the DLRC is now in use across the website and members' platform.

## 4 Discussion

This co‐design study as part of a Participatory Action Research study aimed to design a CoP with people with personal and professional experiences, expertise or interest in dementia, by identifying their preferences for elements within the community, domain and practice. Participatory Action Research using co‐design workshops was chosen so that participants could articulate their needs and preferences for the CoP through a series of workshops [22]. This CoP, the DLRC, was designed to provide a virtual platform to unite people with expertise or interest in dementia, to share knowledge, experiences and insights that enhance understanding, care practices and research in Australia. The Community follows the lead of the Liverpool Dementia & Ageing Research Forum in bringing together people with personal and professional experience of dementia, to break down siloes, empower people living with dementia and current and former carers, and provide informal learning for health and aged care workers and researchers [20]. To the best of our knowledge, this is the first CoP of its kind in published research literature in Australia that brings together both people with personal and professional experiences of dementia for these purposes, and only the second across the globe.

One aspect of the DLRC which differs from the Liverpool Dementia & Ageing Research Forum is that it will take place almost exclusively online. Australia is a large country, and those living in rural and remote areas have limited access to support and care services [24, 25]. We have opted for a virtual platform to create a community that is widely geographically accessible. While there may be some limitations in accessibility for those living with dementia and those with internet connectivity issues, both the Public Advisory Committee and participants in the workshops agreed that a virtual platform provides greater accessibility. Wenger et al. notes that virtual communities are becoming more common and that face‐to‐face meetings are not always necessary for a CoP to function [8]. The use of virtual CoPs for healthcare professionals has been shown to support professional and interprofessional learning and collaborative thinking, break down professional siloes and isolation and create a sense of community [26]. Additionally, virtual CoPs for informal carers of people living with Alzheimer's disease increased social connection and removed time and location constraints [16].

The focus on research and learning in the DLRC was identified in discussions with the Public Advisory Committee in the grant writing stage. Workshop participants confirmed this preference, expressing an interest in learning about all types of dementia research and hearing from dementia researchers. CoPs are based in social learning theory and as such, learning, knowledge gain and knowledge sharing are commonly identified goals [8, 12, 14, 27]. Similarly, CoPs for people with personal experience of dementia have identified knowledge sharing as a desired outcome or purpose, including improved health literacy, learning more about dementia as a disease and support from healthcare professionals to enable learning [16, 17, 18]. Members of the Liverpool Dementia & Ageing Research Forum with personal experience of dementia felt empowered from gaining knowledge and being involved in research [20]. Those from professional backgrounds also benefit from knowledge sharing, identifying improvement of clinical outcomes and sharing evidence‐based practice as key outcomes of CoPs [14, 17, 27]. Bringing together those with personal and professional experience assists in learning for both groups by providing access to evidence‐based knowledge for those seeking information, and allowing access to personal stories to improve real world understanding for those in the workforce [20].

Participants expressed preferences for discussion forums, webinars, panel presentations and smaller group discussions. Wenger et al. [8] highlighted the benefits of having activities which include the whole of a community alongside the webs of relationships that can form between participants. The Liverpool Dementia & Ageing Research Forum similarly provides a variety of activities for participants to attend [15, 20]. Participants stated the importance of having opportunities to connect in smaller groups as a way to get to know other members, and create a comfortable space to share stories and feel heard. Romero‐Mas et al. [16] similarly identified a sense of trust as an important dimension for CoPs for carers of people living with dementia.

### 4.1 Strengths and Limitations

This co‐design activity had some strengths and limitations. While there was an even spread of current and former carers of a person living with dementia, healthcare workers and researchers, the study was limited by the underrepresentation of people living with dementia and aged care workers. This may have caused a bias in the topics and types of activities chosen by participants. It is hoped that through a wider network of recruitment, the members of the DLRC will be more diverse and representative of those living with dementia and those working in the aged care sector. Additionally, other demographics of participants were not collected in this co‐design activity, for example geographical location or gender of participants. This could have similarly caused a bias in the preferred topics, activities and structure of the community. As the community is Australia‐wide, it is important to reach people from rural and remote areas to ensure it is widely accessible. Recruitment of members for the DLRC will aim to be wide reaching to capture those from geographically diverse locations. Additionally, having the workshops and other materials in English limits the participation of culturally and linguistically diverse (CALD) participants. Participation of CALD communities in research is important for creating accessible research outcomes. However, genuine and effective inclusion of CALD communities requires additional time and resourcing. In future, additional resources may be required to provide more accessibility to CALD participants in the DLRC.

This study benefitted from input from a Public Advisory Committee consisting of people with personal experiences of dementia in the preparatory phase of the project, the study design and writing this manuscript [28]. Additionally, the co‐design workshops involved people with different personal and professional experiences of dementia. Despite the limitations of the study, working with these groups of people with personal and professional experience means that the platform is likely to initially meet users' expectations and needs.

### 4.2 Future Directions

A key principle of CoPs is their ability to remain ‘alive’ and be redesigned according to member preferences [8]. While participants were able to express their preferences for the community, domain and practice at this stage, it is possible that these preferences will change over time. Additionally, the community is likely to see a diversity of members who were not represented in this co‐design activity who may have different preferences. The DLRC is expected to evolve and we will undertake regular evaluations with members to gather their feedback and ensure it continues to meet their preferences.

## 5 Conclusion

The DLRC aims to provide a platform for sharing knowledge and information with people with personal and professional experience of or interest in dementia, to break down siloes and enhance understanding of research and care practices. This co‐design activity has brought together people from a range of personal and professional experience backgrounds to identify preferences for the elements of this CoP, to ensure that those who will be a part of the DLRC have shaped it. In future, in‐depth evaluations of the DLRC will be conducted to understand how it is functioning, benefitting attendees and to identify areas for improvement.

## Author Contributions

**Eliza Watson:** investigation, methodology, project administration, writing – original draft preparation, writing – review and editing. **Sadia Afrin:** investigation, writing – review and editing. **Clarissa Giebel:** conceptualisation, methodology, writing – review and editing. **Katrina M. Long:** writing – review and editing. **Jane Thompson:** conceptualisation, methodology, writing – review and editing. **Matthew F:** conceptualisation, methodology, writing – review and editing. **Chris Moran:** conceptualisation, methodology, writing – review and editing. **Yen Ying Lim:** conceptualisation, methodology. **Darshini Ayton:** conceptualisation, funding acquisition, investigation, methodology, project administration, supervision, writing – original draft preparation, writing – review and editing.

## Ethics Statement

Ethics approval was obtained from Monash University Human Research Ethics Committee (project ID 42316) before commencement of the project.

## Conflicts of Interest

The authors declare no conflicts of interest.

## Supporting information

Supplementary material.

## Data Availability

The data that support the findings of this study are not available due to privacy and ethical restrictions.

## References

[1] S. Brand and S. Timmons , “Knowledge Sharing to Support Long‐Term Condition Self‐Management—Patient and Health‐Care Professional Perspectives,” Health Expectations 24, no. 2 (2021): 628–637. 33547706 10.1111/hex.13209 PMC8077082

[2] M. Ray , “Redressing the Balance? The Participation of Older People in Research,” in Critical Perspectives on Ageing Societies, eds. M. Bernard and T. Scharf (Bristol University Press, 2007), 73–88.

[3] R. Tuijt , J. Rees , R. Frost , et al., “Exploring How Triads of People Living With Dementia, Carers and Health Care Professionals Function in Dementia Health Care: A Systematic Qualitative Review and Thematic Synthesis,” Dementia 20, no. 3 (2021): 1080–1104. 32212862 10.1177/1471301220915068 PMC8047709

[4] D. Gove , A. Diaz‐Ponce , J. Georges , et al., “Alzheimer Europe's Position on Involving People With Dementia in Research Through PPI (Patient and Public Involvement),” Aging & Mental Health 22, no. 6 (2018): 723–729, 10.1080/13607863.2017.1317334. 28513210

[5] A. Diaz , D. Gove , M. Nelson , et al., “Conducting Public Involvement in Dementia Research: The Contribution of the European Working Group of People With Dementia to the ROADMAP Project,” Health Expectations 24, no. 3 (2021): 757–765, 10.1111/hex.13246.. 33822448 PMC8235878

[6] J. Miah , S. Parsons , K. Lovell , B. Starling , I. Leroi , and P. Dawes , “Impact of Involving People With Dementia and Their Care Partners in Research: A Qualitative Study,” BMJ Open 10, no. 10 (2020): e039321, 10.1136/bmjopen-2020-039321. PMC7592301 33109666

[7] J. Waite , F. Poland , and G. Charlesworth , “Facilitators and Barriers to Co‐Research by People With Dementia and Academic Researchers: Findings From a Qualitative Study,” Health Expectations 22, no. 4 (August 2019): 761–771, 10.1111/hex.12891. 31012214 PMC6737841

[8] E. Wenger , R. A. McDermott , and W. Snyder , Cultivating Communities of Practice: A Guide to Managing Knowledge (Harvard Business Review Press, 2002).

[9] W. M. Snyder and E. Wenger , “Our World as a Learning System: A Communities‐of‐Practice Approach,” in Social Learning Systems and Communities of Practice, ed. C. Blackmore (Springer London, 2010), 107–124.

[10] L. C. Li , J. M. Grimshaw , C. Nielsen , M. Judd , P. C. Coyte , and I. D. Graham , “Use of Communities of Practice in Business and Health Care Sectors: A Systematic Review,” Implementation Science 4, no. 1 (2009): 27, 10.1186/1748-5908-4-27. 19445723 PMC2694761

[11] J. Lave and E. Wenger , Situated Learning: Legitimate Peripheral Participation. Learning in Doing (Cambridge University Press, 1991).

[12] E. Wenger , Communities of Practice: Learning, Meaning, and Identity. Learning in Doing (Cambridge University Press, 1998).

[13] J. G. Gullick and S. H. West , “Building Research Capacity and Productivity Among Advanced Practice Nurses: An Evaluation of the Community of Practice Model,” Journal of Advanced Nursing 72, no. 3 (2016): 605–619, 10.1111/jan.12850. 26537181

[14] G. Ranmuthugala , J. J. Plumb , F. C. Cunningham , A. Georgiou , J. I. Westbrook , and J. Braithwaite , “How and Why Are Communities of Practice Established in the Healthcare Sector? A Systematic Review of the Literature,” BMC Health Services Research 11, no. 1 (2011): 273, 10.1186/1472-6963-11-273. 21999305 PMC3219728

[15] C. Giebel , H. Tetlow , T. Faulkner , and R. Eley , “A Community of Practice to Increase Education and Collaboration in Dementia and Ageing Research and Care: The Liverpool Dementia & Ageing Research Forum,” Health Expectations 26, no. 5 (October 2023): 1977–1985, 10.1111/hex.13806. 37357808 PMC10485324

[16] M. Romero‐Mas , B. Gómez‐Zúñiga , A. M. Cox , and A. Ramon‐Aribau , “Designing Virtual Communities of Practice for Informal Caregivers of Alzheimer's Patients: An Integrative Review,” Health Informatics Journal 26, no. 4 (December 2020): 2976–2991, 10.1177/1460458220950883. 32951497

[17] S. Lukic , C. Lethin , J. Christensen , and A. Malmgren Fänge , “Reflexive Views of Virtual Communities of Practice Among Informal and Formal Caregivers of People With a Dementia Disease,” Healthcare 12, no. 13 (June 2024): 1285, 10.3390/healthcare12131285. 38998820 PMC11241351

[18] J. D. S. Dedzoe , A. Malmgren Fänge , J. Christensen , and C. Lethin , “Collaborative Learning Through a Virtual Community of Practice in Dementia Care Support: A Scoping Review,” Healthcare 11, no. 5 (February 2023): 692, 10.3390/healthcare11050692. 36900696 PMC10001025

[19] C. Giebel, “Liverpool Dementia & Ageing Research Forum,” https://liverpooldementiaageingresearchforum.co.uk/. 10.1111/hex.13806 PMC10485324 37357808

[20] C. Giebel , J. Watson , M. Polden , M. Readman , H. Tetlow , and M. Gabbay , “Engaging With a Community of Practice in Dementia: Impacts on Skills, Knowledge, Networks and Accessing Support,” Health Expectations 28, no. 1 (February 2025): e70154, 10.1111/hex.70154. 39815691 PMC11735733

[21] W. M. Synder and E. Wenger , “Our World as a Learning System: A Communities‐of‐Practice Approach,” in Social Learning Systems and Communities of Practice, eds. C. Blackmore, (Springer London, 2010). 1st ed.

[22] F. Cornish , N. Breton , U. Moreno‐Tabarez , et al., “Participatory Action Research,” Nature Reviews Methods Primers 3, no. 1 (2023): 34, 10.1038/s43586-023-00214-1.

[23] “Dementia Learning & Research Community. Dementia Learning & Research Community,” accessed April 29, 2025, https://www.dlrc.org.au/.

[24] H. Gulline , S. Carmody , M. Yates , et al., “Equity of Access in Rural and Metropolitan Dementia Diagnosis, Management, and Care Experiences: An Exploratory Qualitative Study,” International Journal for Equity in Health 24, no. 1 (2025): 74. 40091013 10.1186/s12939-025-02434-1 PMC11912628

[25] M. Bauer , D. Fetherstonhaugh , I. Blackberry , J. Farmer , and C. Wilding , “Identifying Support Needs to Improve Rural Dementia Services for People With Dementia and Their Carers: A Consultation Study in Victoria, Australia,” Australian Journal of Rural Health 27, no. 1 (2019): 22–27. 30719789 10.1111/ajr.12444

[26] C. McLoughlin , K. D. Patel , T. O'Callaghan , and S. Reeves , “The Use of Virtual Communities of Practice to Improve Interprofessional Collaboration and Education: Findings From an Integrated Review,” Journal of Interprofessional Care 32, no. 2 (March 2018): 136–142, 10.1080/13561820.2017.1377692. 29161155

[27] A. P. Noar , H. E. Jeffery , H. Subbiah Ponniah , and U. Jaffer , “The Aims and Effectiveness of Communities of Practice in Healthcare: A Systematic Review,” PLoS One 18, no. 10 (2023): e0292343, 10.1371/journal.pone.0292343. 37815986 PMC10564133

[28] N. D. Shippee , J. P. Domecq Garces , G. J. Prutsky Lopez , et al., “Patient and Service User Engagement in Research: A Systematic Review and Synthesized Framework,” Health Expectations 18, no. 5 (2015): 1151–1166, 10.1111/hex.12090. 23731468 PMC5060820

