# Supplementary material for: Co‐Design of a Community of Practice for People With Personal and Professional Expertise or Interest in Dementia in Australia
Source: Health Expect. 2026 Feb 26;29(2):e70503. doi: 10.1111/hex.70503 (PMC12946508; doi:10.1111/hex.70503)
Supplement: Supplementary file 1 — Supplementary material. [file HEX-29-e70503-s001.docx]

**Supplementary material 1**

***Dementia Learning & Research Community – Community Etiquette***

**We are Respectful**

- We are respectful and will listen to what we each have to say.
- We understand that it is ok to respectfully disagree with others.
- We have different abilities, views and experiences and will respect these differences.

**We communicate in many ways**

- We can choose the way to communicate that is easiest for us.
- We can raise our hands, unmute ourselves or use the chat function.
- We understand that some may need more time and space than others to share.

**We are all learning**

- We will be open-minded to listen and learn from others’ views.
- We might make mistakes while we are learning.
- We aim to be understanding of others’ mistakes

**If you ever feel uncomfortable, let the team know.**
